# Prevalence and Perspectives of Complementary and Alternative Medicine among University Students in Atlanta, Newcastle upon Tyne, and New Delhi

**DOI:** 10.1155/2016/9309534

**Published:** 2016-05-03

**Authors:** Kritika Subramanian, Inuka Midha

**Affiliations:** ^1^Northumbria University, Newcastle upon Tyne NE1 8ST, UK; ^2^Georgia Gwinnett College, Atlanta, GA 30043, USA

## Abstract

*Objective*. A cross-cultural comparative study was developed that surveyed university students in Atlanta (United States), New Delhi (India), and Newcastle upon Tyne (United Kingdom) to understand the prevalence and perspectives of CAM in three urban societies with different healthcare systems.* Design*. Surveys were sent to students in the three aforementioned cities. Survey distribution occurred over 6 months from May to November 2015. A total of 314 surveys were received.* Results*. Dietary and vitamin supplements had the highest prevalence collectively (*n* = 203), followed by meditation, yoga, and massage. Commentary analysis showed the importance of science and evidence in justifying CAM practice.* Conclusions*. Matching the most prevalent practices with their designated NCCAM categories suggested that the students were attracted to biologically based, body-based, and mind-body practices as the central themes of attraction. Selected and prevalent CAM practices suggested the students' desire to maintain physical and mental fitness. Access to healthcare may have influence on the prevalence of CAM. Indian students were more likely to view CAM as a viable alternative to conventional medicine.

## 1. Introduction

Complementary and alternative medicine (CAM) is a broad field of medical “therapies” outside the mainstream practice of hospitals. Recently there had been an increase in interest among Americans in CAM. One study conducted in 2007 by the Centers for Disease Control and Prevention (CDC) showed that approximately 38% of Americans (across age groups) had used some sort of CAM practice [1]. A 2015 report by the CDC analyzed trends on CAM usage from 2002 to 2012 among a sample of 88,962 adults [2]. The report depicted a rise in overall CAM prevalence from 2002 to 2007 (32.3% and 35.5%, resp.), followed by a decrease of 2.3% from 2007 to 2012. However, the report showed a growth in Yoga usage with an increase in prevalence from 5.1% in 2002 to 6.1% in 2007 and then 9.5% in 2012. Decrease in CAM usages between 2007 and 2012 was partially attributed to decline in the usage of glucosamine, chondroitin, and combination pill (0.7% decrease) and Echinacea (1.3% decrease). Meditation prevalence also declined from 9.4% in 2007 to 8% in 2012. Chiropractic and osteopathic care changed from 7.5% prevalence in 2002 to 8.6% in 2007 and to 8.4% in 2012. The decline in CAM usage was seen in demographic populations who intuitively should favor CAM, including adults who were not high school graduates, had a salary below the poverty line, and were uninsured [2]. The decreasing popularity of CAM in the United States may be explained by the high level of skepticism on whether the practices actually work: were they supported by science [3, 4]?

CAM usage was often associated with treatment for back pain, depression, insomnia, severe headache/migraine, and gastrointestinal illness. Expectedly, medical students were the greatest CAM skeptics and tended to be the least likely to seek CAM practitioner consultation [5]. This skepticism may be due to a deficiency in the traditional medical curriculum. 78.4% of the medical students in Ireland thought CAM knowledge is important for their medical career. 65% of them thought they have not acquired enough training on CAM therapies in their medical school curriculum, yet only 50.2% thought CAM should be incorporated in the medical curriculum, preferably in the preclinical years [6].

The prevalence of CAM use among American military professionals over 12 months was 44.5% [7]. Interestingly, prayer was included as a form of CAM and 24.4% of the military professionals surveyed acknowledged praying for their own health. 14.1% used massage therapy and 10.8% used a form of relaxation. Comparing these percentages with formerly collected data amongst civilian use, they determined that military personnel used CAM stress therapies 2.5–7 times more often than civilians [7], suggesting that profession influences CAM use.

CAM practices were classified into five categories by the National Center for Complementary and Alternative Medicine (NCCAM). These categories were (1) whole medical systems (e.g., homeopathy and naturopathy), (2) mind-body medicine (e.g., yoga and meditation), (3) biologically based practices (e.g., dietary supplements and herbal remedies), (4) manipulative and body-based practices (e.g., chiropractic medicine, osteopathic medicine, and massage), and (5) energy medicine (e.g., qigong and reiki) [8]. The different types of CAM therapies were designated to fit one or more of the classification “domains.” Domains were defined to include therapies with similar philosophies, purpose, practical training, and individual capability (e.g., routinely practicing yoga or tai-chi in the morning alone) [8].

The most recent large-scale and easily accessible online CAM prevalence report completed in the United States by the CDC in 2007 revealed that the majority of CAM users purchased herbal products, followed by increased usage of deep breathing exercises, yoga, meditation, and massage [9]. These select practices promote physical and mental fitness. Although the 2007 study found that individuals who were concerned about healthcare costs were more likely to use CAM, the same study showed that affluent females were the most likely demographic group to use CAM [9]. Thus, there had to be an attractant aside from cost.

This was supported by the prevalence of CAM usage in countries with socialist healthcare systems where out-of-pocket expenses were necessary to access CAM therapies. For example, a 2010 survey of 7630 individuals in England reported that 44% of the respondents were lifetime CAM users and 26.3% reported a 1-year prevalence [10]. Posadzki et al. did a systematic review of CAM usage in the UK using 5 databases from 2000–2011 [11]. They analyzed 89 surveys covering 97,222 participants. They determined that one-year prevalence of CAM on average was 41.1% and the lifetime prevalence was 51.8%. The greatest average one-year prevalence was for herbal medicines at 64.2% and the greatest lifetime prevalence was for homeopathy at 70%. Forty-one surveys considered the perceived effectiveness of the CAM interventions and, on average, this was calculated to be 49.7% [11]. Harris et al. analyzed 51 reports, 49 of which were surveys, from 15 countries with socialist and private healthcare systems from 1998 and determined that there was significant CAM usage in each of the countries analyzed [12].

The attraction to CAM was undetermined because neither of the CDC reports analyzed what individuals particularly liked about their practice and why they continued (if they did) to practice it [9]. Many private studies have attempted to answer this question within a demographic group. McFadden et al. surveyed 65 graduate students of psychology and found positive correlations between CAM usage and individuals who believed in the CAM philosophy, were disappointed with conventional medicine, and/or supported holistic care [13]. However, limitations in this study included the education and gender bias (72% of respondents were female [13]), preventing the application of its findings to a larger, more diverse student population.

Other studies which analyzed CAM opinions and usage looked specifically into the influence of medical education on the opinion of students in the UK [14, 15], Australia [16], and the US [17, 18] or compared the opinions of students in a particular region of Australia [19], Canada [20], and the US [21, 22]. Again, these studies showed a discipline bias (most were medical students) or a regional bias. One way to overcome regional and discipline bias would be to perform a study on CAM opinions in different cities with a random selection of students.

Our study surveyed university students in Atlanta (US), New Delhi (India), and Newcastle upon Tyne (UK) to understand the prevalence and perspectives, or the positions and views they hold, of CAM in three culturally different, urban societies with different healthcare systems. The alternative hypothesis was that the usage of CAM was based on access to healthcare and the desire to practice preventative medicine through fitness techniques. The specific cities were chosen due to accessibility to reach out to students via listservs. The 2007 National Institute of Health (NIH) survey analysis of CAM use in the United States [9] showed an increase in yoga, meditation, and herbal supplements, offering credible consideration to add a South Asian city in the analysis. The presence of a socialist healthcare system in the UK provided support for including a British city in the analysis to consider the role of cost and access to healthcare system relative to CAM usage. Comparing opinions of CAM across cultures helped to pinpoint tentative CAM attractants to university students worldwide.

## 2. Methods

A structured, anonymous survey was spread online via email listservs for participation. University students of any age, gender, and discipline were asked to participate. Based on a confidence interval of 10 and a confidence level of 95% for a maximum student population size of 100,000 the goal was set to receive at least 100 completed surveys from each city. The survey was written using Google Forms and presented to all takers in an informal, nonobligatory manner ensuring anonymity. In accordance with the Ethical Review Board (ERB) approval, a statement at the beginning of the survey informed the participants that the survey responses were meant for the purpose of research and publication.

Aside from basic demographic details and the presence of comment/opinion boxes, the survey asked the students to mark any CAM practice they have tried/used at least once in their life based on a list of examples from Johns Hopkins Medicine Health Library's website [23]. The survey questions were adjusted to match the equivalent practice of the surveyed country. For example, the UK survey asked whether or not the student was pleased with the way the National Health Service (NHS) worked instead of saying the UK Healthcare System. Survey Distribution occurred over 6 months, from May to November 2015.

## 3. Results

Three hundred and fourteen completed surveys were received from all the three cities: exactly 100 valid survey responses from Atlanta (US), 111 from New Delhi (India), and 103 from Newcastle upon Tyne (UK). Individual responses were compared with the time of submission to avoid duplication of any survey entered. Any survey that was submitted twice was deleted. Data analysis was done using Microsoft Excel. Chi-square analysis and *p* value calculations were conducted using the online program GraphPad to compare bivariate responses within a city (Table 4). One-way ANOVA analysis was done using the online program Vassarstats and SPSS to compare results between the cities and was followed by Chi-square tests to determine which cities were significantly different from one another.

Respondents were not asked for their age in the survey. However, the fact that the responses came from a wide range of ages can be deduced from the degree and discipline they studied (Tables 1 and 2). 32.5% of these respondents were male (*n* = 102) and 67.5% of the respondents were female (*n* = 212).

**Figure 1:**
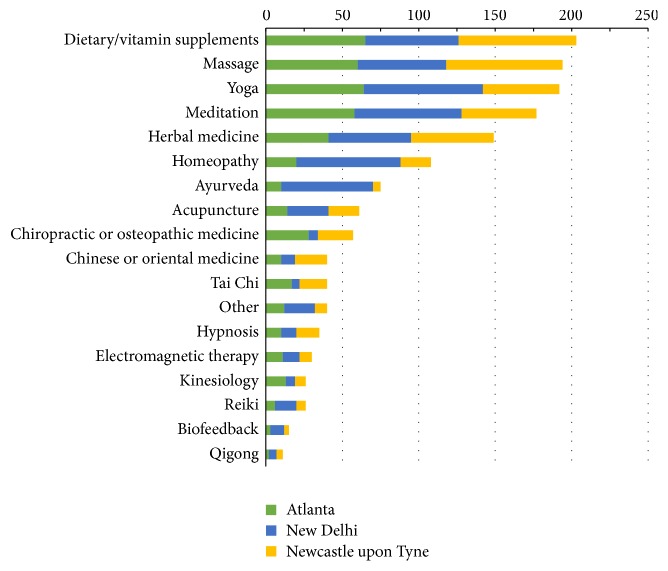
Prevalence (observed number) of CAM therapies in each city. Positive responses include individuals who regularly practice the therapy and those who have attempted or tried it once.

Chi-square and *p* values were measured comparing individual city prevalence with the overall prevalence findings. Analyzing the CAM prevalence in each individual city showed that, in Atlanta and Newcastle upon Tyne, over 50% of the respondents had tried dietary and vitamin supplements at least once (Table 3). Meditation, yoga, herbal medicine, and massage (all with *n* > 40) were also highly prevalent in this population of respondents. The greatest number of New Delhi respondents had tried yoga and meditation, closely followed by massage, herbal medicine, homeopathy, dietary and vitamin supplements, and Ayurveda (all with *n* > 50). CAM practices that were the greatest in prevalence overall included dietary and vitamin supplements massage, yoga, and meditation (Figure 1). Table 4 depicts the proportion of one-time CAM users relative to more frequency users in each surveyed city.

One-way ANOVA analysis combined with the Tukey HSD test found direct correlation between answering patterns on the questionnaire: did a certain response to one question correlate with a certain response to another? Individuals who thought CAM incorporation would make healthcare more affordable and those who believed CAM should be integrated in the healthcare system were more likely to have used home remedies, used CAM at least once in their life, or thought CAM was beneficial. This correlation was the strongest in the New Delhi responses. However, ANOVA and Tukey HSD analysis did not show any correlation between the answers of individuals who thought CAM incorporation would make healthcare more affordable with the answers of individuals who thought CAM should be integrated in the healthcare system.

One-way ANOVA analysis also showed that comparison between cities was significant for whether or not the students were satisfied with their healthcare system (*p* = 0.015) and the usage of CAM to address a health concern (*p* < 0.001). Newcastle upon Tyne had the greatest percentage of satisfaction with the healthcare system. Gender correlated with CAM usage such that females were more likely to try homeopathy, massage, yoga, herbal medicine, meditation, and dietary or vitamin supplements. Females were also more likely to find CAM to be beneficial (*p* < 0.001). However, bias could explain this finding due to the greater number of female respondents (67.5% of total responses).

A significant number of students in Atlanta and New Delhi used home remedies. Both these groups were generally dissatisfied with their local healthcare system. The majority of students from Newcastle upon Tyne did not think CAM should be integrated in the healthcare system and believed CAM incorporation will not make healthcare more affordable. On the other hand, students from New Delhi thought CAM incorporation will make healthcare more affordable. The majority of students in all three cities had used CAM at least once in their lives and did not believe that CAM could substitute allopathic medicine (Table 5). Out of 289 students who had tried CAM at least once in their life, 26.6% used it in response to a health concern they had (*n* = 77, *X* ^2^ = 81.529, *p* < 0.0001). In general, the study illustrated that the majority of respondents have tried CAM but not because of a clinical diagnosis.

## 4. Commentary Analysis

Of the comments (*n* = 13) left by the New Delhi students, 84.6% were positive opinions (*X* ^2^ = 49.00, *p* < 0.0001). Out of the positive responses, 54.5% of the comments shared a common theme of holistic care, whole-body care, and over all well-being as their reason for trusting CAM.

Survey comments from Newcastle upon Tyne agreed that CAM was useful for minor treatments and stated that CAM was good alongside allopathic medicine (*X* ^2^ = 5.143, *p* = 0.0233, 28.6% of respondents for *n* = 28). 64.3% of the responses were generally positive. 25% of respondents mentioned science or the need for CAM to “work” through evidence-based medicine (*X* ^2^ = 7.00, *p* = 0.0082). A few responses were highly supportive of CAM, including the mention of CAM being an alternative to paracetamol and antibiotics.

The Atlanta survey comments (*n* = 11) were dispersed, where CAM opinions ranged from it being a scam and meant for desperate people to CAM being good for temporary relief. The latter opinion was shared by 54.5% of the comments that supported the use of certain CAM therapies. 80% of the negative responses shared the opinion that if CAM therapies were effective and supported by science, then they would be called medicine and not alternative medicine (*X* ^2^ = 7.840, *p* < 0.0051). One commenter typed a paragraph on why cancer patients should decline chemotherapy, reasoning that the best treatment for cancer was to increase oxygen perfusion by eating well.

A combined analysis of the survey responses (*n* = 52) from the three surveyed cities indicated that CAM could be accepted as part of medical treatment if it worked effectively on patients (25%, *X* ^2^ = 6.760, *p* = 0.0093). 67.3% were positive CAM opinions (*X* ^2^ = 6.231, *p* = 0.0126). 23.1% of the comments indicated the need for science and evidence-based practice of complementary medicine (*X* ^2^ = 15.077, *p* < 0.0001).

## 5. Discussion

Dietary and vitamin supplements, yoga, meditation, and massage were the most prevalent CAM therapies in all three cities. Matching these practices with their designated NCCAM categories suggested that the overall theme university students were attracted to was biologically based, body-based, and mind-body practices. However, it was very much possible that the students were aware of nutritional requirements and were taking supplements to address those concerns [24]. This reasoning was more acceptable than the suggestion that students were taking supplements because they believed in the power of nature as a therapeutic intervention. Body-based and mind-body interventions were believed to promote self-awareness and self-care. Students and young adults were likely to take advantage of these practices in order to destress and reduce anxiety [25, 26].

Whole medical systems (Ayurveda and homeopathy) were relatively prevalent among the Indian students, supplementing the common theme of holistic and whole-body care from the survey comments of Indian participants. The high prevalence of Ayurveda, a form of traditional medicine, and other whole medical systems, followed by the belief in holistic care, suggested that many of these students may have chosen one form of medicine over the other. Homeopathy, believed to carry a placebo effect [27], was an interesting finding on the perceptions of medicinal drugs and their usage in India. In 2010, there were 200,000 registered homeopathic doctors and they were believed to be increasing every year by 12,000 [28]. Many Indian students viewed CAM as an acceptable alternative form of medicine (35%). 55% of the Indian students believed that incorporating CAM into the healthcare system would make healthcare more affordable. Therefore, the possibility of cost as a major attractant could not be ruled out in this population. It would be worth noting that despite the healthcare system in India being mostly private, only 6% of the population have medical insurance in the country [29]. Most Indians incurred out-of-pocket expenses to pay for their healthcare [29].

Energy medicine was not popular in any of the three cities surveyed. This could be due to a suspected lack of survey takers from East Asian heritage. Although the survey asked what the ethnicity of each respondent was, East Asians and South Asians were categorized together as “Asians,” thereby preventing further analysis.

Understanding the purpose of CAM usage among a demographic population would assist in the integration of CAM with conventional medicine. Contrary to widespread belief, simultaneous use of CAM in conventional medicine may not be far-fetched. The student responses from this study showed that proponents of CAM used the therapies to improve their physical or mental health. It is important to understand to what extent is that belief medically acceptable? Studies have shown that the incorporation of CAM in the conventional treatment regimen of chronic illnesses was correlated with an improvement in patient quality of life [30, 31]. La Cour observed that a significant proportion of rheumatoid arthritis patients did not tell their physicians of their CAM usage. The 15 elderly patients they interviewed used CAM therapies because they believed that “natural is best” and not because they viewed CAM as a form of medicine [32]. This perspective of CAM as a medical supplement might explain why the patients did not disclose their use of CAM to their physicians. The same idea was seen in the survey responses where 75% of respondents did not view CAM as an alternative to allopathic medicine. Survey comments also reiterated that CAM practices that worked were just called “medicine,” suggesting that most university students did not view CAM practices as medical therapies.

The popularity of certain CAM therapies in certain countries or ethnic populations can be attributed to cultural factors, for example, the popularity of Ayurveda amongst the Indian students. Cultural factors such as religion, language, and the name given to a plant as well as its interpretative meaning have been shown to influence individual acceptance of CAM therapy, specifically herbal medicine [33]. In cultures where physicians were more likely to be religious, including Indonesia and India, they were also more likely to feel comfortable in addressing the spiritual needs of their patients. These cultures showed more integration of complementary and alternative medicine in their allopathic treatment [34]. In certain countries, including Turkey and other Asian countries, traditional medicine was incorporated into the religious and local culture centuries ago, the history of which offered a comfort unfound in allopathic care [35]. CAM prevalence was also correlated with the GDP of a country such that low-income countries showed a higher prevalence of CAM and traditional medicine [36]. Cultural perspectives of CAM therapies in these countries include the ability of CAM to be effective, yet harmless, holistic, and still progressive. Traditional Chinese medicine and Ayurveda attract clients by individualizing the medical regime [37]. These cultural factors explain why India had the greatest CAM acceptance rate relative to the UK and US in the findings. CAM modalities such as yoga and meditation are popular in the western countries not only due to the proportion of immigrants residing there but also due to the fact that there are no negative side effects and are therefore more readily accepted. A 2012 National Health Interview Survey Analysis (*n* = 34,525) suggested that CAM users in the United States were more likely to use the complimentary health approaches (specifically yoga and natural product supplement users) for wellness-related reasons, rather than for medical treatment. These wellness-related reasons included disease prevention, improved immunity, better energy, holistic outlook, and improved memory or concentration [38].

The findings that certain CAM therapies may strengthen the immunity have led to the incorporation of CAM in mainstream allopathic medicine with tentative therapeutic benefit. Reddy et al. searched for beneficial CAM products on PubMed and requested American dermatologists and surgeons to use them in their clinical practice perioperatively [39]. Analysis of the results showed potential benefits in the use of bromelain, honey, propolis, Amica, vitamin C and bioflavonoids, chamomile, and others. The benefits included promotion of wound healing, reduction in edema or purpura, and anti-inflammatory effects. They also suggested that use of these CAM products has the risk to cause platelet inhibition, contact dermatitis, and systemic toxicity, though such side effects are rare [39].

Comparing the prevalence results from the student responses with the responses from studies looking at different communities helps determine popular CAM therapies relative to medical health status, beliefs for use of CAM, and age. Recently, there has been a surplus of research analyzing the use of CAM among cancer survivors. From the 2007 National Health Interview Survey (*n* = 23,393), 66.5% of the 1,471 cancer survivors reported CAM usage to enhance their immune system and prevent disease. The prevalence of CAM among this subgroup of the survey respondents was attributed to the recommendation of the reporting healthcare provider [40]. Similar studies among cancer patients conducted on an international, multinational scale showed 40% prevalence rate in CAM, the greatest usage being in the US and the lowest in Italy and the Netherlands [41]. The CAM therapies more likely used by CAM survivors were yoga, meditation or mindfulness, energy heeling, medical qigong, homeopathy, and mistletoe therapy [42].

In Germany, 500 outpatient children were surveyed and the results showed that 57% of them were CAM users, most commonly homeopathy (25%), herbal remedies (8%), and anthroposophic medicine (7%). These therapies were selected by the parents of the pediatric patients to strengthen their children's immunity and to maintain physical and mental health, similar reasons to what was seen in the collective university student analysis [43].

## 6. Conclusion

Student interest in promoting self-care and preventative medicine could be seen in their selection and usage of CAM therapies. Although some students tried CAM to address a health concern they had, the majority used CAM for other reasons. In India, CAM has a financial advantage of lower cost than conventional medicine. But in Western countries with socialized healthcare systems and prevalent medical insurance policies, CAM practices have a higher out-of-pocket expense [44, 45]. However, the more prevalent CAM practices were those that cost less than others including dietary and vitamin supplements, yoga, and meditation. “Integrative Medicine” [46], a subspecialty under Family or Internal Medicine, was an evidence-based, holistic approach of alternative medicine that was being integrated in the conventional practice. Many American hospitals have included a separate division for Integrative Medicine, including the Duke Center for Integrative Medicine and the Arizona Center for Integrative Medicine, a result of the popularity and ongoing research on complementary and alternative medicine therapies. Integrative Medicine was a mechanism to reduce healthcare costs, promote preventive medicine and self-care, augment cultural competency amongst physicians, and recommend patients with chronic or terminal illness to allopathic specialists at the correct times in their own hospital setting. It would be interesting to analyze CAM usage trends in hospitals which offer Integrative Medicine therapies. Future research should also compare the cost of each CAM therapy with its usage.

## Figures and Tables

**Table 1:** Percentage of respondents belonging to each discipline of study. 1% of respondents did not answer this question.

Discipline of study	%
Humanities	12.1
Social sciences	15.9
Life and health sciences	38.9
Law	2.5
Engineering	5.7
Mathematics and computer science	3.8
Other	20.1

**Table 2:** Percentage of respondents belonging to each degree program. 0.5% of respondents did not answer this question.

Degree program	%
Associate's degree	2.9
Bachelor's degree	54.5
Master's degree	15
Doctoral degree	22.3
Other	4.8

**Table 3:** Statistical values for the prevalence of the top 5 most popular CAM therapies.

		*n* (sample size)	*X* ^2^	*p* value
Dietary and vitamin supplements	Atlanta	65	9	0.0027
	Newcastle upon Tyne	77	26.255	<0.0001
	Delhi	61	1.297	0.2547
	Composite	203	26.955	<0.0001

Massage	Atlanta	60	4	0.0455
	Newcastle upon Tyne	76	24.274	<0.001
	Delhi	58	0.324	0.569
	Composite	194	17.439	<0.0001

Yoga	Atlanta	64	7.84	0.0051
	Newcastle upon Tyne	50	0.039	0.8438
	Delhi	78	19.065	<0.0001
	Composite	192	15.605	<0.0001

Meditation	Atlanta	58	2.56	0.1096
	Newcastle upon Tyne	49	0.155	0.6935
	Delhi	70	8.109	0.0044
	Composite	177	5.096	0.024

Herbal medicine	Atlanta	41	3.24	0.0719
	Newcastle upon Tyne	54	0.35	0.5544
	Delhi	54	0.036	0.8494
	Composite	149	0.815	0.366

**Table 4:** Percentage of one-time CAM users with total prevalence.

	Composite		Atlanta		New Delhi		Newcastle upon Tyne	
	Once (%)	Total (%)	Once (%)	Total (%)	Once (%)	Total (%)	Once (%)	Total (%)
Acupuncture	10.1	21.9	7.3	14.6	12.3	33.3	10.9	19.8
Ayurveda	3.6	26.7	1	10.3	9.5	1.4	1	5
Homeopathy	6.6	37.6	1	20.8	15.6	75.6	4	19.8
Chinese or oriental medicine	2.6	14.6	1.1	10.5	2.5	11.1	4.1	21.4
Chiropractic or DO	5.5	20.4	6.3	29.5	3.8	7.5	6	22
Massage	12.8	68.4	15.6	62.5	11	68.2	10.9	74.3
Tai Chi	5.8	14.5	6.3	17.9	1.3	6.3	9	18
Yoga	9.3	66.1	10.5	67.4	9.7	83.9	7.9	48.5
Herbal medicine	6.8	53.4	4.2	43.2	8.3	64.3	8	54
Electromagnetic therapy	5.5	11.7	3.2	11.7	8.8	16.3	5	8
Kinesiology	3.3	9.5	4.3	13.8	1.2	7.4	4	7
Reiki	3.6	9.5	1.1	6.3	9.9	17.3	1	6.1
Qigong	1.5	4.4	0	2.1	2.5	7.5	2	4
Meditation	10.6	62.8	8.4	61.1	9.2	80.5	14	49
Hypnosis	6.5	12.7	6.3	10.5	5	12.5	8	15
Biofeedback	1.8	5.5	1.1	3.2	3.8	11.4	1	3
Dietary or vitamin supplement	7.1	72.2	5.3	68.4	11.9	72.6	4.9	75.5
Other	3.6	18.1	2.5	15	6.3	31.7	2.6	10.3

**Table 5:** Comparison of the observed number and percentage (in parentheses) of responses from each city to bivariable question analysis. Positive response values are given in the table. The remaining responses (1 − positive response percentage) reflect negative responses, uncertainty, and no response. *p* values that are in bold are statistically significant (*p* < 0.05). Percentage values were rounded to the closest whole number to reflect how the values were entered on GraphPad. Calculations were made on 2 × 1 contingencies tables with a 50% expected value if *H*_0_ (no difference between the positive and negative responses) is true for a binary analysis. The *p* values are comparing positive response percentages with negative response percentages within the same city to determine if there is any significant opinion that could be correlated with the culture of the city. Collective *p* values were calculated using ANOVA analysis.

		Atlanta	New Delhi	Newcastle upon Tyne	Collective
Gender	Male	30 (31%)	40 (36%)	32 (31%)	102 (33%)
	Female	70 (69%)	71 (64%)	71 (69%)	212 (68%)

Used home remedies		68 (68%)	91 (82%)	61 (60%)	220 (70%)
	*p* value	**0.0003**	**<0.0001**	0.0719	**<0.0001**

Thought CAM is beneficial		54 (54%)	63 (57%)	53 (52%)	170 (54%)
	*p* value	0.4237	0.1615	0.6892	0.4237

Were satisfied with local healthcare system		15 (15%)	8 (7%)	46 (45%)	69 (22%)
	*p* value	**<0.0001**	**<0.0001**	0.3173	**<0.0001**

Thought CAM can substitute allopathic medicine		22 (22%)	39 (35%)	16 (16%)	77 (25%)
	*p* value	**<0.0001**	**0.0027**	**<0.0001**	**<0.0001**

Thought CAM should be integrated in the healthcare system		51 (51%)	63 (57%)	38 (37%)	152 (48%)
	*p* value	0.8415	0.1615	**0.0093**	0.6892

Thought CAM incorporation will make healthcare more affordable		52 (52%)	61 (55%)	33 (32%)	146 (46%)
	*p* value	0.6892	**0.0278**	**0.0003**	0.4237

Used CAM at least once in their life		88 (88%)	102 (92%)	99 (96%)	289 (92%)
	*p* value	**<0.0001**	**<0.0001**	**<0.0001**	**<0.0001**

## Additional Points

Even though the surveys were distributed through online platforms to receive a completely random selection of responses, sample bias can appear when friends discuss the survey. Students of less-intensive study disciplines have more time on their hands to complete a survey than students of other disciplines which may also have contributed to bias. Gender bias was present because of the unintentional greater number of female respondents. When comparing CAM usage between groups, many ANOVA analyses would show *p* < 0.05 but when performing unpaired *t*-tests for individual pairs, the results would be insignificant. This discrepancy is most likely a result of the small sample size of the individual CAM practice (e.g., acupuncture had only 14 positive responses in Atlanta, 27 in New Delhi, and 20 in Newcastle upon Tyne).
